# Detection and frequency of recombination in tomato-infecting begomoviruses of South and Southeast Asia

**DOI:** 10.1186/1743-422X-4-111

**Published:** 2007-10-26

**Authors:** HC Prasanna, Mathura Rai

**Affiliations:** 1 Indian Institute of Vegetable Research, P B 5002, P 0-B H U, Varanasi, Uttar Pradesh, 221005, India

## Abstract

**Background:**

Tomato-infecting begomoviruses are widely distributed across the world and cause diseases of high economic impact on wide range of agriculturally important crops. Though recombination plays a pivotal role in diversification and evolution of these viruses, it is currently unknown whether there are differences in the number and quality of recombination events amongst different tomato-infecting begomovirus species. To examine this we sought to characterize the recombination events, estimate the frequency of recombination, and map recombination hotspots in tomato-infecting begomoviruses of South and Southeast Asia.

**Results:**

Different methods used for recombination breakpoint analysis provided strong evidence for presence of recombination events in majority of the sequences analyzed. However, there was a clear evidence for absence or low Recombination events in viruses reported from North India. In addition, we provide evidence for non-random distribution of recombination events with the highest frequency of recombination being mapped in the portion of the N-terminal portion of Rep.

**Conclusion:**

The variable recombination observed in these viruses signified that all begomoviruses are not equally prone to recombination. Distribution of recombination hotspots was found to be reliant on the relatedness of the genomic region involved in the exchange. Overall the frequency of phylogenetic violations and number of recombination events decreased with increasing parental sequence diversity. These findings provide valuable new information for understanding the diversity and evolution of tomato-infecting begomoviruses in Asia.

## Background

Begomoviruses are an important group of whitefly (*Bemisia tabaci*) transmitted viruses in the family *Geminiviridae*. They inflict significant economic losses in many dicotyledonous crops including beans, cassava, cotton, melon, pepper, potato and tomato [1-7]. Tomato yellow leaf curl virus (TYLCV) and Tomato leaf curl virus (ToLCV) are the begomoviruses severely constraining tomato production in many tomato-growing regions of the world.

Begomovirus genomes are composed of either one (monopartite) or two (bipartite) single stranded DNA molecules ranging in size between 2500 and 2800 nucleotides [8]. Most TYLCV of the old world and almost all known new world begomoviruses viruses are bipartite with genomes comprising DNA A and DNA B molecules. Monopartite old world begomoviruses, which are now believed to be the predominant begomovirus form, have only a DNA-A like genome component. The virion-sense strand of DNA A encodes the viral coat protein (AV1, V1 or *cp*) and, in old-world begomoviruses [9], an AV2 or V2 gene that is necessary for virus accumulation and symptom development [10]. The complementary-sense strand of DNA-A encodes genes responsible for viral replication (AC1, C1 or *rep*), replication enhancer (AC3, C3 or *ren*), regulation of gene expression (AC2, C2 or *trap*) and AC4 or C4 involved in host range determination, symptom determination, symptom severity, and virus movement [11-13]. The DNA B of bipartite begomoviruses encodes two proteins, BV1 (a nuclear shuttle protein or NS) and BC1 (a movement protein or MP) involved in intra- and inter-cellular movement within the plant [14].

Begomoviruses exhibit a great deal of geographic dependent but host-independent genomic variation [15-17]. Recombination, especially interspecific homologous recombination, is a key contributor to the genomic diversification and evolution of begomoviruses [17]. To date, many natural begomoviruses recombinants have been reported [17-20]. Although the biological significance of begomovirus recombination is not clearly understood, in many parts of the world epidemics associated with the emergence of recombinant begomoviruses have been reported. These include the devastating cassava mosaic disease epidemic caused by recombinant East African cassava mosaic viruses in Uganda and neighbouring countries [18,21], the currently emerging pathogenic recombinant, tomato yellow leaf curl Malaga virus, in Spain [22] and the cotton leaf curl disease epidemic in Pakistan caused by a species complex including a variety of mostly recombinant begomovirus species [23]. Besides the apparent importance of recombination in begomovirus evolution the marks that it has left on currently sampled begomovirus genome sequences also have major implications when we attempt to use these sequences to infer the evolutionary histories of begomoviruses [24,25]. Consequently, the detailed characterization of recombination amongst tomato-infecting begomoviruses is a prerequisite for understanding how these important pathogens are evolving.

Although a few specific recombination events have been described so far in tomato-infecting begomoviruses [26-29], a full accounting of recombinants, recombination breakpoints and recombination hotspots in tomato begomovirus species and strains is lacking. For example, it is currently unknown whether there are differences in the number and quality of recombination events that are occurring amongst different tomato infecting begomovirus species. It is also currently unknown whether sequences in particular parts of the begomovirus genomes are more or less exchangeable between different species than sequences in other parts of these genomes. Such variations in recombination frequencies and patterns have been clearly observed in RNA viruses [30]. In this study we employ a variety of recombination analysis methods to characterize recombination in South and Southeast Asian tomato-infecting begomoviruses. We map recombination hotspots and provide evidence that not all tomato-infecting begomoviruses are equally prone to recombination and that specific characteristic of particular recombination events are reliant on both the relatedness of the recombining viruses and the genomic region involved in sequence exchanges

## Results and discussion

In this study, we sought to characterise recombination in South and Southeast Asian viruses using a different approach to those used previously: (1) By studying a different set of viruses to those studied previously; (2) Making use of a combination of recombination analysis methods that are both powerful and have low false positive rates; (3) by mapping and estimating the frequency of recombination events in begomoviruses.

**Figure 1:**
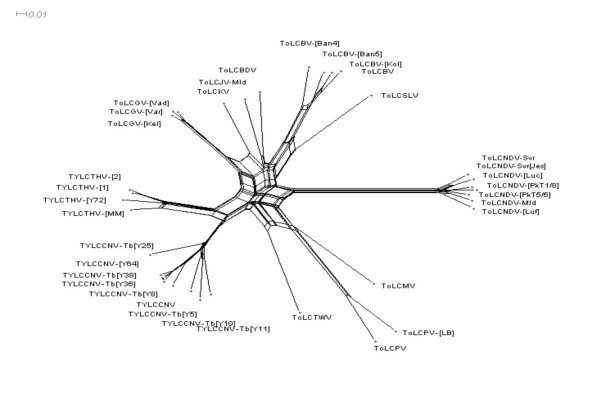
**Neighbor-Net generated for the tomato-infecting begomoviruses of South and Southeast Asia.** Evidence for reticulate evolution is reported on pairwise Hamming distances using only parsimonious sites. Networked relationships among the viral species with boxes, instead of bifurcating evolutionary tree indicate to the presence of recombination.

The neighbor-net analysis revealed clear evidence of phylogenetic conflicts within the analysed sequences (Fig. 1). Notably, every sequence represented within the tree was implicated as a potential recipient of horizontally acquired sequences at some time in its evolutionary past. Unsurprisingly, the PHI test strongly supported the presence of recombination in these sequences (p < 0.0001).

**Figure 2:**
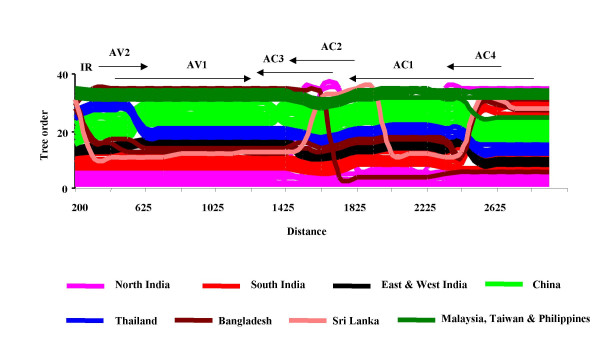
TreeOrder Scan of tomato-infecting begomoviruses sequences. Changes in tree order(Y axis) resulting from changes in phylogenetic relationships at 70% bootstrap level are shown for sequential 300 bases sequence fragments at 100 base fragment intervals (X axis). Sequences are assigned to groups based on geographical locations and groups are color coded as indicated by labels. The genome map drawn to scale has been superimposed to indicate the positions of genes in DNA A sequences. Positions were drawn relative to the ToLCGV-[Var] strain.

Different methods used for recombination breakpoint analysis also provided strong evidence for presence of past recombination events in most of the sequences analysed. For each of the 32 potential recombinant sequences identified, possible breakpoint positions, sequence fragments and parental genotypes are listed in Table 1. Tomato leaf curl virus from the Philippines and ToLCBV, ToLCBV-[Ban4] and ToLCBV-[Ban5] from Bangalore, south India appeared to be the most complex recombinants carrying evidence of seven and six recombination events respectively. On the opposite end of the spectrum, Tomato leaf curl virus strains including ToLCNDV-Mld and ToLCNDV-[Luc] from New Delhi, ToLCNDV-Svr [Jes] from Bangladesh, and TYLCCNV-Tb [Y38] from China each carried evidence of only a single recombination event. In addition, viruses from geographically well separated regions appeared to have recombined at some time in the past. For example, tomato leaf curl virus strains from Bangalore and Gujarat in India contained sequences closely resembling those found in a ToLCTWV isolate from Taiwan. Also, Chinese viruses contained fragments of sequence closely resembling those found in sequences sampled in Thailand, Taiwan, Bangladesh and South India. Further, we used the TreeOrderScan method [31] to investigate the phylogenetic evidence for recombination in the sequence alignment. This analysis revealed major deviations in the branching order of sequences within trees constructed from different portions of the multiple sequence alignment (Fig. 2). Frequent tree order changes were observed at the region of rep and AC4. Importantly, most of the viruses detected as recombinants in the breakpoint analysis exhibited deviations in their branching order indicating that they were most likely correctly identified as recombinants. In addition, the TreeOrderScan analysis also provided evidence for gene flow amongst viruses in geographically separated regions. For instance, sequences found in southern Indian viruses grouped with those found in Thailand and Bangladesh virus positions from 2335–2652. Thai viruses contained sequences resembling those of Chinese viruses between 300–490 and 590–2372, but Indian viruses between 2472–2743. The recombination observed between geographically separated species/strains probably represents older events as they presumably occurred before their present separation [19]. Movement of vectors and/or infected plant materials may also have contributed to the gene flow observed between these widely separated locations [32]. Alternatively, it is possible that current sampling of Asian begomovirus diversity is so sparse that we do not yet fully appreciate the geographical range of many of the species studied here.

**Table 1:** Breakpoint analysis of tomato-infecting begomoviruses and their putative parental sequences.

Species/Strain	ORF	Breakpoint	Possible parent	Method*
ToLCNDV-Mld	AC1	2119–2211	Unknown	gc, rdp
ToLCNDV-Svr [Jes]	AC1, AC4	1953–2507	ToLCPV-[LB]	RDP, gc
ToLCNDV-[Luc]	AC1, AC4	1960–2514	ToLCPV	RDP, gc
ToLCBV-[Ban4]	AV1, AV2	481–894	ToLCKV	GC, rdp. mc
	AC1, AC2, AC3	1185–1784	Unknown	gc
	AC1	1793–1894	Unknown	rdp
	AC1, AC4	2076–2632	ToLCGV-[Vad]	RDP
			ToLCGV-[Var]	GC
			ToLCGV-[Kel]	MC
ToLCBV-[Ban5]	AV1, AV2	481–894	ToLCKV	GC, rdp
	AC1, AC2, AC3	1185–1784	Unknown	gc
	AC1	1793–1894	Unknown	rdp, mc
		2579–2708	ToLCBDV	GC, MC, rdp
	AC1, AC4	2144–2734	ToLCTWV	GC
		2183–2376	ToLCTWV	RDP
ToLCBV-[Kol]	AV1, AV2	481–894	Unknown	rdp
	AC1, AC2, AC3	1185–1784	Unknown	gc
	AC1	1793–1894	Unknown	rdp
	AC1, AC4	2144–2727	ToLCTWV	GC
		2183–2376	ToLCTWV	RDP
ToLCBV	AV1, AV2	481–894	ToLCKV	RDP, GC
	AC1, AC2, AC3	1185–1784	Unknown	gc
	AC1	1793–1894	Unknown	rdp
		2585–2623	ToLCPV-[LB]	RDP
	AC1, AC4	2141–2724	ToLCTWV	RDP
		2180–2374	ToLCTWV	GC
ToLCGV-[Kel]	AV1, AV3	598–1214	TYLCTHV-[Y72]	RDP, gc
			TYLCTHV-[1]	MC
	AC1, AC2, AC3	1183–1782	Unknown	gc
	AC1, AC4	2160–2514	ToLCTWV	RDP, GC
ToLCGV-[Var]	AV1, AV3	603–1219	TYLCTHV-[Y72]	RDP, gc
			TYLCTHV-[1]	MC
	AC1, AC2, AC3	1188–1787	Unknown	gc
	AC1, AC4	2165–2519	ToLCTWV	GC
ToLCGV-[Vad]	AV1, AV3	598–1214	TYLCTHV-[1]	RDP, MC, gc
	AC1, AC2, AC3	1183–1782	Unknown	gc, mc
	AC1, AC4	2160–2514	ToLCTWV	RDP, GC
TYLCCNV-Tb [Y36]	AV1, AV2	451–924	ToLCPV-[LB]	RDP, gc
	AC1, AC4	2053–2213	ToLCTWV	GC
TYLCCNV-Tb [Y38]	AV1, AV2	451–924	ToLCPV-[LB]	RDP, gc
TYLCCNV-[Y64]	AV1	525–927	ToLCSLV	RDP, gc
		451–924	Unknown	gc
	AC1, AC4	2053–2213	ToLCTWV	GC, RDP
TYLCCNV-Tb [Y8]	AV1, AV2	451–924	ToLCPV-[LB]	RDP, gc
	AC1, AC4	2051–2210	ToLCTWV	GC, RDP
TYLCCNV	AV1, AV2	455–928	Unknown	rdp, gc
	AC1, AC4	2057–2217	ToLCBV-[Kol]	GC, RDP
TYLCCNV-Tb [Y10]	AV1, AV2	450–923	Unknown	gc, rdp
	AC1, AC4	2044–2482	TYLCTHV-[MM]	RDP, GC
TYLCCNV-Tb [Y11]	AV1, AV2	450–923	Unknown	rdp. gc
	AC1, AC4	2044–2482	ToLCTWV	RDP
			TYLCTHV-[MM]	GC
TYLCTHV-[2]	AV1, AV2	296–1197	Unknown	rdp, gc
	AC1, AC4	2200–2360	ToLCBDV	GC
		2390–2630	ToLCTWV	RDP, GC
TYLCTHV-[1]	AV1, AV2	305–1206	Unknown	rdp, gc
	AC1, AC4	2203–2363	ToLCBDV	GC
		2393–2633	ToLCTWV	RDP, GC
TYLCTHV-[MM]	AV1, AV2	157–1058	Unknown	rdp, gc
		2058–2218	ToLCBV-[Kol]	GC
TYLCTHV-[Y72]	AV1, AV2	158–1059	Unknown	rdp, gc
	AC1	2059–2219	ToLCBV	GC
	AC1, AC4	2249–2489	ToLCTWV	RDP
			TYLCCNV-Tb [Y10]	GC
TYLCCNV-Tb [Y5]	AV1	451–924	Unknown	Gc
	AC1	2051–2210	ToLCBV	GC
TYLCCNV-Tb [Y25]	AC1	2054–2214	ToLCTWV	RDP, GC
		2487–2661	Unknown	Mc
ToLCKV	AV1	708–875	ToLCBV-[Ban5]	RDP, GC
	AC1, AC2, AC3	1182–1781	Unknown	gc, mc
	AC1, AC4	2159–2513	ToLCTWV	RDP, GC
ToLCJV-Mld	AC1, AC2, AC3	1184–1783	Unknown	gc, mc
	AC1, AC4	2143–2736	TYLCCNV-Tb [Y5]	RDP
			TYLCCNV-Tb [Y25]	GC
		2058–2321	ToLCBV-[Kol]	GC
		2588–2642	TYLCCNV-Tb [Y5]	GC
ToLCBDV	AC1, AC2, AC3	1184–1783	Unknown	gc, mc
	AC1, AC4	2058–2321	ToLCTWV	RDP
			ToLCBV-[Kol]	GC
		2143–2735	Unknown	rdp, gc
ToLCSLV	AV1, AV2	132–467	ToLCBV-[Ban4]	GC, MC
	AC1, AC2, AC3	1097–1608	Unknown	rdp, gc
	AC1	1789–1890	Unknown	rdp, mc
	AC1, AC4	2140–2731	ToLCTWV	RDP, gc, mc
ToLCMV	IR	2687–34	TYLCCNV-Tb [Y25]	RDP
		2687–38	TYLCCNV	RDP
		2687–61	Unknown	Mc
ToLCPV	AC1, IR	2537–77	TYLCCNV	RDP
	AV2, IR	2727–78	Unknown	Rdp
	IR, AV2	39–68	Unknown	gc, mc
		0–70	Unknown	Gc
	IR	2715–43	ToLCPV-[LB]	MC
	AC1, AC4	2306–2627	TYLCCNV-Tb [Y11]	GC, mc
	AC1	2508–2715	Unknown	rdp, gc, mc
ToLCPV-[LB]	AC1, AC4	2162–2685	Unknown	rdp, gc
ToLCTWV	AC1	2494–2671	Unknown	gc, mc

*The method in upper case is for the method identifying putative parent

Interestingly, our breakpoint analysis indicated that three north Indian viruses (ToLCNDV-[PkT1/8], ToLCNDV-Svr and ToLCNDV-[PkT5/6]) were not detectably recombinant and three other north Indian viruses namely ToLCNDV-Mld, ToLCNDV-[Luc] and ToLCNDV-[Luf] were simple recombinants with only evidence of a single detectable recombination event involving a virus resembling ToLCPV sampled in the Philippines. While TreeOrderScan analysis also revealed an absence of recombination in two north Indian viruses, ToLCNDV-[PkT1/8] and ToLCNDV-[Luf] (indicated by a horizontal line across the graph in Fig. 2). In addition, there was no phylogenetic support for inter-group recombination event reported for ToLCNDV-[Luc]. Thus there appears to be no or few recombination events in viruses reported from North India, signifying that certain begomovirus species may not recombine as readily as others. There are a number of prerequisites for recombination between begomoviruses. These include shared host ranges (possibly influenced by the emergence of B whitefly biotype), the ability to co-infect the same cells [33-35], high levels of viral replication [36], and overlapping geographical ranges. If all of these prerequisites are met for the tomato-infecting begomoviruses in South and Southeast Asia then one would expect there to be frequent and invariable recombination amongst all of these viruses. However, fitness disadvantages may be associated with some sequence exchanges that would lead to the selective elimination of many newly produced recombinants.

The recombination sites distributed non-randomly along the genome. The recombination breakpoints were detected in all the six reading frames of south Indian viruses and viruses from eastern and western India. The breakpoints in the Chinese and Thai viruses were located in AV1, AV2, AC1 and AC4, whereas ORFs AV1 and AV2 were identified to be cold spots in the Bangladeshi viruses.

**Figure 3:**
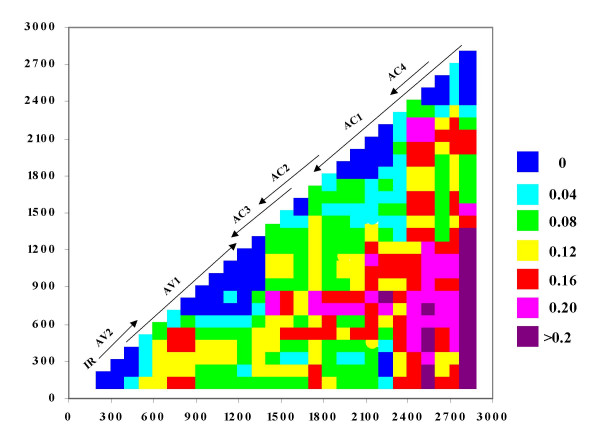
Phylogenetic compatibility matrix of tomato-infecting begomovirus sequences, exhibiting frequencies of phylogeny violations for each pairwise comparison of sequence fragments. For this analysis sequence fragments of 300 bases and 100 base intervals were used. Phylogeny violations above the threshold bootstrap value of 70% are shown. Frequencies are color coded to indicate number of phylogeny violations per sequence. The genome map drawn to scale has been superimposed to indicate the positions of genes in DNA A sequences. Positions were drawn relative to the ToLCGV-[Var] strain.

The frequency and locations of recombination events measured as topological differences between trees constructed from different parts of the alignment were visualised as a half-diagonal compatibility matrix (Fig. 3). Each X and Y coordinate in the matrix is a gross estimate of the number of topological modifications needed to convert the tree constructed using sequences at position X into that constructed using sequences at position Y [31,37]. It was apparent from this matrix that recombination events are probably not randomly distributed throughout begomovirus genomes. The highest frequency of recombination apparently occurs in the portion of the C1/AC1 ORF encoding the N-terminal portion of Rep. For example, the matrix indicates that there are an excess of 0.16 phylogeny violations per clade when trees constructed using sequences between alignment positions 351 and 1251 are compared with those constructed using sequences between alignment positions 2451 and 2951. This analysis also indicated the probable absence in certain regions of begomovirus genomes of recombination events that had any substantial phylogenetic effect. For example, all phylogenetic trees constructed using coat protein gene sequences were all in good agreement with one another indicating a relative absence of recombination breakpoints within the CP gene.

**Figure 4:**
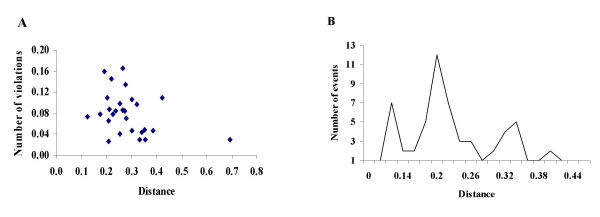
(A) Relationship between the number of phylogeny violations and fragment diversity. Jukes-Cantor distance was calculated for each pairwise comparison used in TreeOrder Scan analysis and corresponding violations were counted and plotted. (B) Relationship between the number of recombination events and fragment diversity. The fragments involved in the exchange with identified parental sequences were used and the number of recombination events detected were counted and plotted.

We examined phylogeny violations and number of recombination events in our data set from the perspective of parental sequence relatedness. We noted that in general phylogeny violations clustered around the genetic distance 0.30. The observed frequency of phylogeny violations were inversely correlated (r = -0.36 p < 0.05) to the pairwise distances of the fragments involved in exchange (Fig. 4A). In addition, the number of recombination events was also inversely correlated (r = -0.35 p < 0.05) to the diversity between the exchanged fragments (Fig. 4B), we used only identified parental sequences to estimate the genetic distance between horizontally transferred fragments and the sequences that they replaced. Overall the frequency of phylogenetic violations and number of recombination events decreased with increasing parental sequence diversity. In a study with artificial and natural geminivirus recombinants Martin and co-workers [38] demonstrated that the degree of similarity between a horizontally inherited sequence and the sequence it replaces is an important determining factor of recombinant fitness. Rather than the non-random distribution of break points observed here being due to higher recombination rates in some genome regions than others [39], the distribution seems to have been created by natural selection only allowing the survival of recombinants with high fitness. In the more diverse genome regions where recombination events are not detected it is possible that these regions would not function properly when transferred into foreign genetic backgrounds.

## Conclusion

Finally, the variable recombination and diversity-dependent distribution of recombination hotspots in tomato-infecting begomoviruses is valuable new information that has emerged from this study. Perhaps this is the first report of variable recombination reported among tomato-infecting begomoviruses found in the same region. Further, recombinant forms, recombination hot spots and frequency of recombination documented in this study would provide new information for understanding the diversity and evolution of tomato-infecting begomoviruses in Asia. In addition to evolutionary considerations, understanding the implications of recombination observed in these viruses on efforts to develop resistant tomatoes through conventional breeding and genetic engineering are important and attempts should be focused on these issues for developing effective disease management strategies. Given that the N-terminal portion of rep is highly recombinogenic it is perhaps worrying that so many virus derived transgenic resistance strategies are focusing on this portion of the geminivirus genome [40-43]. It may be wiser to develop virus derived resistance strategies using genome regions that are less recombinogenic as this will make it more difficult for viruses to overcome resistance by simply replacing targeted genome regions with variants that are not targeted.

## Methods

### Sequence data

The study sequences comprised 35 publically available (as on June 2006) complete Indian, Pakistani, Chinese, Bangladeshi, Sri Lankan, Malaysian, Thai, Philippine and Taiwanese tomato-infecting begomovirus DNA-A and DNA-A-like components (Table 2). These sequences were aligned using the CLUSTAL W [44] using gap open and extension penalties- of 10.

**Table 2:** List of species/strains of tomato-infecting begomoviruses used in the present study.

Species/strain	Genbank accession	Abbreviation
Tomato leaf curl Bangalore virus	Z48182	ToLCBV
Tomato leaf curl Bangalore virus-[Ban4]	AF165098	ToLCBV-[Ban4]
Tomato leaf curl Bangalore virus-[Ban5]	AF295401	ToLCBV-[Ban5]
Tomato leaf curl Bangalore virus-[Kolar]	AF428255	ToLCBV-[Kol]
Tomato leaf curl Bangladesh virus	AF188481	ToLCBDV
Tomato leaf curl Gujarat virus-[Kelloo]	AF449999	ToLCGV-[Kel]
Tomato leaf curl Gujarat virus-[Vadodara]	AF413671	ToLCGV-[Vad]
Tomato leaf curl Gujarat virus-[Varanasi]	AF190290	ToLCGV-[Var]
Tomato leaf curl Joydebpur virus-Mild	AJ875159	ToLCJV – Mld
Tomato leaf curl Karnataka virus	U38239	ToLCKV
Tomato leaf curl Malaysia virus	AF327436	ToLCMV
Tomato leaf curl New Delhi virus-Mild	U15016	ToLCNDV-Mld
Tomato leaf curl New Delhi virus-Severe	U15015	ToLCNDV-Svr
Tomato leaf curl New Delhi virus-Severe [Jessore]	AJ875157	ToLCNDV-Svr [Jes]
Tomato leaf curl New Delhi virus-[Lucknow]	Y16421	ToLCNDV-[Luc]
Tomato leaf curl New Delhi virus-[Luffa]	AF102276	ToLCNDV-[Luf]
Tomato leaf curl New Delhi virus-[PkT1/8]	AF448059	ToLCNDV-[PkT1/8]
Tomato leaf curl New Delhi virus-[PkT5/6]	AF448058	ToLCNDV-[PkT5/6]
Tomato leaf curl Philippines virus	AB050597	ToLCPV
Tomato leaf curl Philippines virus-[LB]	AF136222	ToLCPV-[LB]
Tomato leaf curl Sri Lanka virus	AF274349	ToLCSLV
Tomato leaf curl Taiwan virus	U88692	ToLCTWV
Tomato yellow leaf curl China virus	AF311734	TYLCCNV
Tomato yellow leaf curl China virus-[Y64]	AJ457823	TYLCCNV-[Y64]
Tomato yellow leaf curl China virus-Tb [Y10]	AJ319675	TYLCCNV-Tb [Y10]
Tomato yellow leaf curl China virus-Tb [Y11]	AJ319676	TYLCCNV-Tb [Y11]
Tomato yellow leaf curl China virus-Tb [Y36]	AJ420316	TYLCCNV-Tb [Y36]
Tomato yellow leaf curl China virus-Tb [Y38]	AJ420317	TYLCCNV-Tb [Y38]
Tomato yellow leaf curl China virus-Tb [Y5]	AJ319674	TYLCCNV-Tb [Y5]
Tomato yellow leaf curl China virus-Tb [Y8]	AJ319677	TYLCCNV-Tb [Y8]
Tomato yellow leaf curl China virus-Tb [Y25]	AJ457985	TYLCCNV-Tb [Y25]
Tomato yellow leaf curl Thailand virus-[1]	X63015	TYLCTHV-[1]
Tomato yellow leaf curl Thailand virus-[2]	AF141922	TYLCTHV-[2]
Tomato yellow leaf curl Thailand virus-[Myanmar]	AF206674	TYLCTHV-[MM]
Tomato yellow leaf curl Thailand virus-[Y72]	AJ495812	TYLCTHV-[Y72]

### Phylogenetic network and pairwise homoplasy test

Phylogenetic evidence for recombination was detected with Splits-Tree version 4.3 [45] using the neighbor-Net method [46]. Neighbor-net depicts conflicting phylogenetic signals in the data that are caused by recombination as cycles within unrooted bifurcating trees. Although, we report evidence for reticulate evolution in such phylogenetic graphs obtained using parsimonious sites, pairwise Hamming distances and no gaps, we obtained similar results with other distance measures and settings.

We statistically verified the presence of recombination identified visually in phylogenetic graphs using the pairwise homoplasy test (PHI) implemented in Splits Tree 4.3. PHI has been shown to powerfully identify the presence/absence of recombination within a wide range of sequence samples with a low false positive rate [47].

### Detection of recombination breakpoints

The recombination breakpoint analysis was carried out using Recombination detection program RDP [48], GENECONV [19] and MAXIMUM CHI SQUARE [49], selected following the conclusions of studies on evaluation of different methods of recombination detection [50,51]. All these methods are implemented in RDP2 [52,53]. Default RDP2 settings were used throughout (P-value cut-off = 0.05 and the standard Bonferroni correction was used), other than that sequences were considered as circular, consensus daughters were found and breakpoints were polished. We used principally the information inferred by more than one method, as evaluation of the performance of these recombination detection methods using simulated and empirical data indicated that one should not rely too heavily on the results of a single method (Posada, 2002). In RDP analysis, the length of the window was set to 10 variable sites, and the step size was set to one nucleotide. P values were estimated by randomizing the alignment 1,000 times. For GENECONV analysis, the g-scale parameter was set to 1 and the number of permutations was set to 10,000.

### Phylogenetic congruence

To examine phylogenetic support for each identified recombination event in the breakpoint analysis, we used the retained sequence position version of the TreeOrder Scan method [31] implemented in Simmonics2005 (Version1.4) package. TreeOrder Scan records the position of each sequence in a series of phylogenetic trees produced by sets of overlapping fragments across the genomes. Deviations in the tree order of individual sequences and of group of sequences between fragments of defined length indicate conflicting phylogenetic relationships. Alternatively, individual non-recombinant sequences show constant tree order (position) across the genome. In the present analysis, we recorded the changes in the phylogenetic relationships of clades supported by 70 per cent bootstrap values for sequential 300 base sequence fragments at 100 nucleotide intervals.

### Frequency and mapping of recombination

Estimation of the frequency and mapping of the locations of recombination events was achieved by phylogeny compatibility analysis using the TreeOrder Scan method. First, the TreeOrder Scan program produces optimally ordered neighbor-joining trees for fragments of definite length along an alignment. In the next step, a pairwise comparison is made between trees constructed from each sequence fragment along the alignment. Then a phylogenetic compatibility value is computed as the number of times the phylogeny of one tree has to be violated to match the tree order observed in other trees constructed along the length of an alignment. In our case we assigned sequences to predefined groups based on their geographical origin and a bootstrap value of 70 per cent was used as threshold for scoring phylogeny violations. All pairwise compatibility values were calculated using trees constructed for 300 nucleotide sequence fragments separated by 100 nucleotides across the length of the analysed alignment. These compatibility values were then plotted on a phylogenetic compatibility matrix.

## Competing interests

The author(s) declare that they have no competing interests.

## Authors' contributions

HCP conceived and designed the study; HCP, MR executed the study and wrote the paper. Both the authors read and approved the final manuscript.
